# Estimation of the number of heat illness patients in eight metropolitan prefectures of Japan: Correlation with ambient temperature and computed thermophysiological responses

**DOI:** 10.3389/fpubh.2023.1061135

**Published:** 2023-02-17

**Authors:** Akito Takada, Sachiko Kodera, Koji Suzuki, Mio Nemoto, Ryusuke Egawa, Hiroyuki Takizawa, Akimasa Hirata

**Affiliations:** ^1^Department of Electrical and Mechanical Engineering, Nagoya Institute of Technology, Nagoya, Japan; ^2^Architecture, Design, Civil Engineering, and Industrial Management Engineering, Nagoya Institute of Technology, Nagoya, Japan; ^3^Department of Environment Systems, Graduate School of Frontier Sciences, The University of Tokyo, Chiba, Japan; ^4^School of Engineering, Tokyo Denki University, Tokyo, Japan; ^5^Cyberscience Center, Tohoku University, Sendai, Japan

**Keywords:** ambient heat, ambulance dispatch, heat adaptation, heat illness, global warming

## Abstract

The number of patients with heat illness transported by ambulance has been gradually increasing due to global warming. In intense heat waves, it is crucial to accurately estimate the number of cases with heat illness for management of medical resources. Ambient temperature is an essential factor with respect to the number of patients with heat illness, although thermophysiological response is a more relevant factor with respect to causing symptoms. In this study, we computed daily maximum core temperature increase and daily total amount of sweating in a test subject using a large-scale, integrated computational method considering the time course of actual ambient conditions as input. The correlation between the number of transported people and their thermophysiological temperature is evaluated in addition to conventional ambient temperature. With the exception of one prefecture, which features a different Köppen climate classification, the number of transported people in the remaining prefectures, with a Köppen climate classification of Cfa, are well estimated using either ambient temperature or computed core temperature increase and daily amount of sweating. For estimation using ambient temperature, an additional two parameters were needed to obtain comparable accuracy. Even using ambient temperature, the number of transported people can be estimated if the parameters are carefully chosen. This finding is practically useful for the management of ambulance allocation on hot days as well as public enlightenment.

## 1. Introduction

Global warming is making heat waves more intensive, longer-lasting, and more common worldwide (1–3). The mortality and mobility of heat-related illness caused by heat waves have been extensively studied (4–7). To assess the impact of further global warming, different climate models have been proposed at the overall warming temperatures of 1.5°C, 2°C, and 3°C (8, 9). According to Song et al. (10), the risk factors for heat-related mortality are different at the global, intermediate, and local scales.

In aging societies, heat-related morbidity and mortality are expected to increase (11). Japan has the highest proportion of the elderly in the world (12). The yearly number of cases of heat-related illness transported by ambulance over the entirety of Japan, with its total population of 125 million, has been increasing gradually, from a range of 40,000 to 60,000 from 2010 to 2017 and then reaching a record high of 95,137 in 2018. Since then, it has remained high, at 60,000–70,000 (13). The number of cases registered with the use of an ambulance is closer to the actual number of cases than is the case in other countries, as ambulance transportation is free in Japan.

A positive correlation has been reported between ambient temperature and the number of heat illness patients (14–16). The surge of transported patients on hot summer days results in a greater ambulance use, causing temporary shortages. Thus, estimating the number of heat illness patients is essential for ambulance allocation management and dynamic systems operation on hot summer days (17).

Heat-related illnesses are broadly classified according to two symptoms, namely, dehydration and collapse of heat balance (18). The thermophysiological parameters for these are water loss and core temperature, respectively, although the two are related. The time course response of thermoregulation is significantly affected by age, lifestyle, and environment, making the epidemiology and pathology of heat stroke difficult to ascertain.

Several methods of estimating numbers of cases of heat-related illness have been proposed using analysis (19, 20) and machine learning taking weather data (21, 22). In Nishimura et al. (20), we demonstrated that estimation using regression models has comparable accuracy to that produced by a machine learning architecture. Our regression model identifies that the weather condition is associated with adverse heat illness events not only on the day of the event but also on preceding days (~3days). This is particularly obvious for non-external heat-related illness in the elderly. Thus, additional efforts are needed to identify external heat-related illness or estimate total number of patients. In particular, the association between population-level estimated core temperature and water loss with the number of transported people is worth evaluating in different regions.

In this study, we developed an estimation model for the number of heat illness patients in eight metropolitan prefectures in Japan. In particular, an integrated computational technique was used that took into account multiphysics and thermophysiology to derive the thermophysiological response of a standard test subject for different weather condition. Heat adaptation at different prefectures is also considered.

## 2. Methods

### 2.1. Data sources

**Figure 1:**
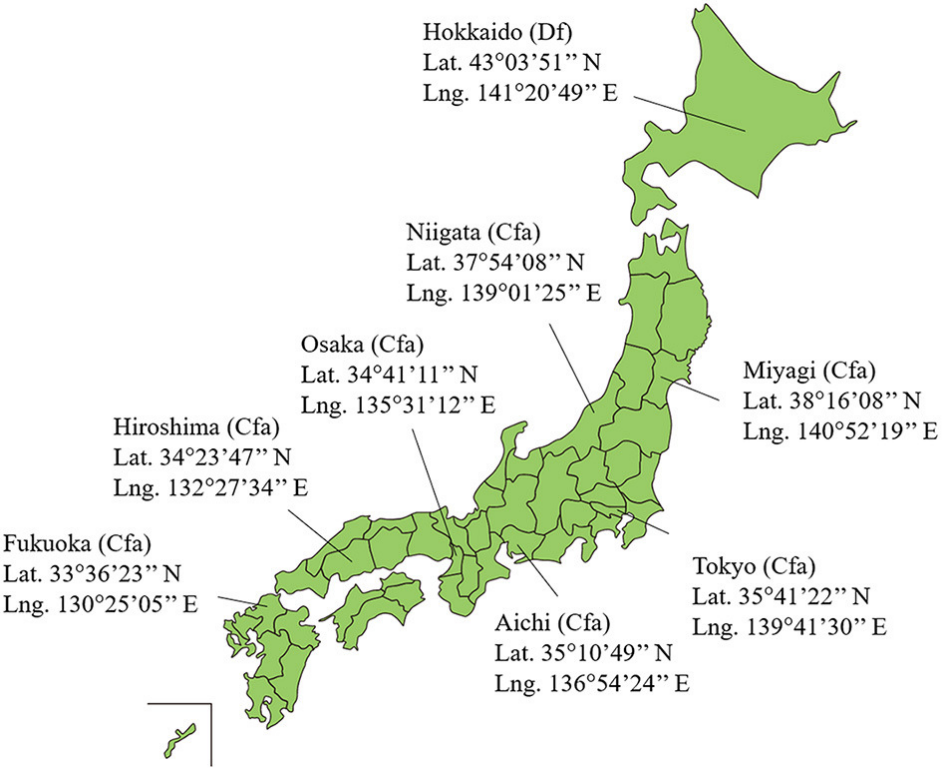
Location of eight prefectures in Japan selected for this study. Japan includes 47 prefectures. The Köppen climate classification in each prefecture are also presented.

Eight prefectures with different climatic conditions were selected for this study, as shown in Figure 1. The northernmost, Hokkaido, is at 43°03'51” N and 141°20'49” E, and the southernmost, Fukuoka, is located at 33°36'23” N and 130°25'05”E. The climates are classified as Df and Cfa within the Köppen scheme (23).

Three datasets were utilized in this study. The first dataset includes the daily ambient temperature provided by Japan Meteorological Agency, Japan (24). The second describes the age composition of the population in each prefecture, as provided by Official Statistics of Japan (25).

The final dataset reports the number of people transported by ambulance owing to heat-related illnesses, provided by the Fire and Disaster Management Agency under the Ministry of Internal Affairs and Communications, Japan, between 2013 and 2019 (13). They provided the daily number of ambulance dispatches owing to heat-related illness in each prefecture from June to September annually. Data regarding the transported patients have been collected by prefectures by date and age categories (infant, child, adult, and elderly) since 2013, and the categories of occurrence location were added from 2017. The number of people transported *via* ambulance due to heat-related illness in Japan can be used as a surrogate marker because the number of transported people and that of the number of patients are correlated with each other (26). The dataset for the number of transportations from 2013 to 2016 that were not classified by occurrence location was substituted by dividing the total number by mean percentage transported from indoor locations/homes and outdoor locations/workplaces from 2017 to 2019.

Table 1 shows the daily average and maximum ambient temperatures per summer from June 1 to September 30 from 2013 to 2019, the average population and the population density from 2013 to 2019, and the average number of heat illness patients per million population per summer from June 1 to September 30 from 2013 to 2019 in each prefecture.

**Table 1:** Daily average and maximum ambient temperature per summer from June 1 to September 30, average population and population density, and average number of patients due to heat-related illness per million population per summer from June 1 to September 30, all data from 2013 to 2019.

	**Average ambient temperature (°C)**	**Maximum ambient temperature (°C)**	**Population (millions)**	**Population density per km^2^**	**Number of patients per million**
Hokkaido	20	33.7	5.3	63.7	192.9
Miyagi	22.4	35.6	2.3	319.1	391.8
Niigata	23.9	36.9	2.3	181.3	497.3
Tokyo	25.1	37.4	13.6	6205	330.5
Aichi	25.9	38.1	7.5	1449.9	506.4
Osaka	26.4	37.8	8.8	4634.6	469.2
Hiroshima	26	36.8	2.8	333.7	529.4
Fukuoka	26.2	37.3	5.1	1022.8	458.9

Table 2 shows the percentage of heat illness patients transported from indoor/home and outdoor/workplace from 2017 to 2019. The variation in the ratio of the transported people from the indoor/home and outdoor/workplace over the 3 years showed a ≤ 8% variation. Unlike a definition of Fire and Disaster Management Agency, places that are not related to their homes were classified as outdoor considering the consistency of non-external heat-related illness in the elderly.

**Table 2:** Percentage of heat illness patients transported from indoor/home and outdoor/workplace from June 1 to September 30 from 2017 to 2019.

**Prefecture**	**Location**	**2017**	**2018**	**2019**	**Average**
Miyagi	Indoor	40.8	41.3	48.0	43.9
	Outdoor	59.2	58.7	52.0	56.1
Niigata	Indoor	40.7	40.7	40.7	40.7
	Outdoor	59.3	59.3	59.3	59.3
Tokyo	Indoor	37.3	40.5	40.3	39.9
	Outdoor	62.7	59.5	59.7	60.1
Aichi	Indoor	32.8	39.0	35.2	36.5
	Outdoor	67.2	61.0	64.8	63.5
Osaka	Indoor	34.5	38.2	32.7	35.6
	Outdoor	65.5	61.8	67.3	64.4
Hiroshima	Indoor	42.5	42.7	43.2	42.8
	Outdoor	57.5	57.3	56.8	57.2
Fukuoka	Indoor	37.0	40.5	38.0	38.7
	Outdoor	63.0	59.5	62.0	61.3

### 2.2. Equation for estimating number of transported patients owing to heat-related illness

In our previous studies (19, 20), we proposed an equation for estimating the number of transported people owing to heat-related illness. In particular, the classification of indoor/home and outdoor/workplace was considered in Nishimura et al. (20), which are approximately surrogate for non-exertional and exertional heat stroke. The number of transported people can be estimated as follows:


(1) $$\begin{array}{lll} y\left(x\right) & = & y_{in}\left(x\right)+y_{out}\left(x\right), \end{array}$$

(2) $$\begin{array}{l} y_{in}\left(x\right)=a_{in}\left[e^{k_{in}(0.6x_{0}+0.2x_{1}+0.2x_{2})}+l_{in}\right]\\ \;\cdot \sum_{n}^{}\left\{P\left(n\right)\cdot \left(be^{cn}+d\right)\right\}, \end{array}$$

(3) $$\begin{array}{lll} y_{out}\left(x\right) & = & a_{out}\left(e^{k_{out}x_{0}}+l_{out}\right)\cdot \underset{n}{\sum }\left\{P\left(n\right)\cdot \left(be^{cn}+d\right)\right\}, \end{array}$$

(4) $$\begin{array}{lll} k_{in,out} & = & f_{in,out}\cdot \left(\overset{J}{\underset{i=1}{\sum }}w_{i}\cdot x_{i}\right)+g_{in,out}, \end{array}$$

where *y*_*in*_ and *y*_*out*_ denote daily heat illness patients transported from indoor/home and outdoor/workplace, respectively. The variable *x*_*i*_ denotes the input variable *i* days ago; *x*_0_ indicates the input variable for the predicted day. The parameters *a, l, f* , and *g* are fitting parameters. Three types of input variable were selected for daily average temperature, daily maximum body core temperature increase, and amount of sweating. Unlike Nishimura et al. (20), the daily maximum body core temperature increase and the amount of sweating were computed using our in-house computational code (Section 2.3).

The number of cases of heat illness with transportation from indoor locations [Equation (2)] is affected by the daily average temperature for three successive days; the weightings of *x*_0_, *x*_1_, and *x*_2_ were 0.6, 0.2, and 0.2, respectively (19), in addition to qualitative discussion in Williams et al. (27). The numbers of patients transported from outdoor [Equation (3)] are affected by the climate on the corresponding day (28). Parameter *n* denotes age category [five-year age intervals; *n* = 1 (20–24 years old), …,14 (85 years old and over)]; *P*(*n*) denotes the age composition of population in each prefecture. The function *be*^*cn*^ + *d* is the regression curve derived in Figure 4 in Kodera et al. (19) expressed the increase in the risk of heat-related illness with age; the parameters *b* (=0.171), *c* (=0.494), and *d* (=190.7) determined by the least-squares fitting method, based on the age components of heat illness patients in Japan.

Equation (4) represents the short-term heat adaption during the summer, i.e., the risk of heat-related illnesses *k* (coefficient of input variable *x*_*i*_) with a decrease from the beginning to the end of summer, affected by climate over the previous several tens of days (29, 30). *J* provides the number of weighting days, and parameter *w*_*i*_ denotes weighted linear function. The input variables and optimal duration of *J* are evaluated in Section 3.2.

Fitting parameters *a, l, f*, and *g* were estimated to provide an expected number of cases of heat illness with transportation from indoor and outdoor locations, respectively. The parameters *a* and *l* were determined first, followed by *f* and *g*. All of the parameters were averaged over a 7-year time frame. Specifically, parameter fitting was iteratively conducted for convergence: 6 years of data (extracted over 7 years of data from 2013 to 2019) were used to determine the parameters, and then those for the remaining year were used through a leave-one-out cross-validation study (31). That is, data from 2013 to 2018 were used to determine the parameters for estimating the number of heat illness patients in 2019. Because the amount of sweating and the increase in body core temperature may be zero on days with a low heat load, we set *l* = −1 when these input variables were used, so that the number of transported patients converged to zero.

The estimation accuracy was evaluated in terms of the determination coefficient (*R*^2^) and mean absolute error (MAE). The F-test was carried out for Equations (2, 3) with each input variable: daily average temperature, daily maximum increase in core temperature, and amount of sweating. All statistical analysis were conducted using Python 3.9.7. The threshold for a statistical significance was set at *p* < 0.05 (see Section 4 in the Supplementary material).

### 2.3. Computation of core temperature and water loss due to sweating

Body core temperature increase and amount of sweating, which were derived by computation, were used as surrogates for estimating the daily number of transported people using the non-linear analysis, in addition to the daily average ambient temperature. Ambient temperature showed a good correlation with the mobility of heat-related illness (14–16). The increase in body core temperature and the amount of sweating were computed in the time domain using our in-house computational code, taking into account the time series of ambient temperature and relative humidity from 2013 to 2019 (see Figure S2) (24).

Our computation combines thermodynamics in biological tissues and thermoregulation, including vasodilatation and sweating due to increased body temperature. Our computational code is summarized in the Section 2 in Supplementary material. To accelerate the computation, the in-house computational code was vectorized and parallelized and subsequently implemented on an SX-Aurora TSUBASA system named AOBA (32). A detailed and validation of our in-house computational code was presented in our previous studies (33, 34).

## 3. Results

### 3.1. Computed body core temperature and sweating

**Figure 2:**
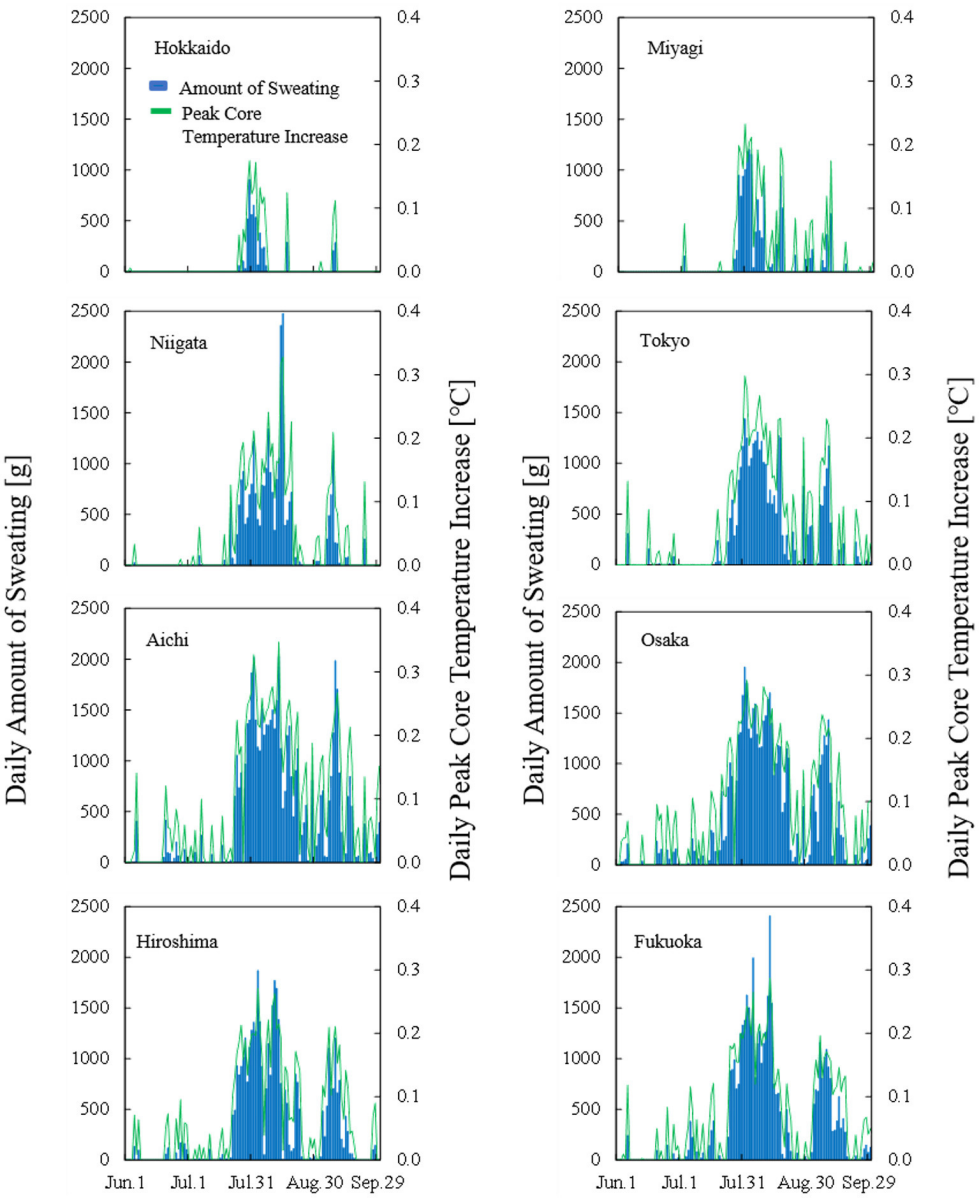
Time course of computed daily peak core temperature and total amount of sweating in each prefecture in 2019.

Figure 2 shows the computed daily peak core temperature and daily amount of sweating for each prefecture in 2019. The thermophysiological response to ambient heat stress was significant in early August across Japan. In Hokkaido, body core temperature increase and sweating amount were low even in August. It should also be noted that the distributions of body core temperature increase and sweating resembled each other. Their correlation coefficient was more than 0.93 (*p* < 0.05) for each prefecture.

### 3.2. Duration of short-term heat adaptation

**Figure 3:**
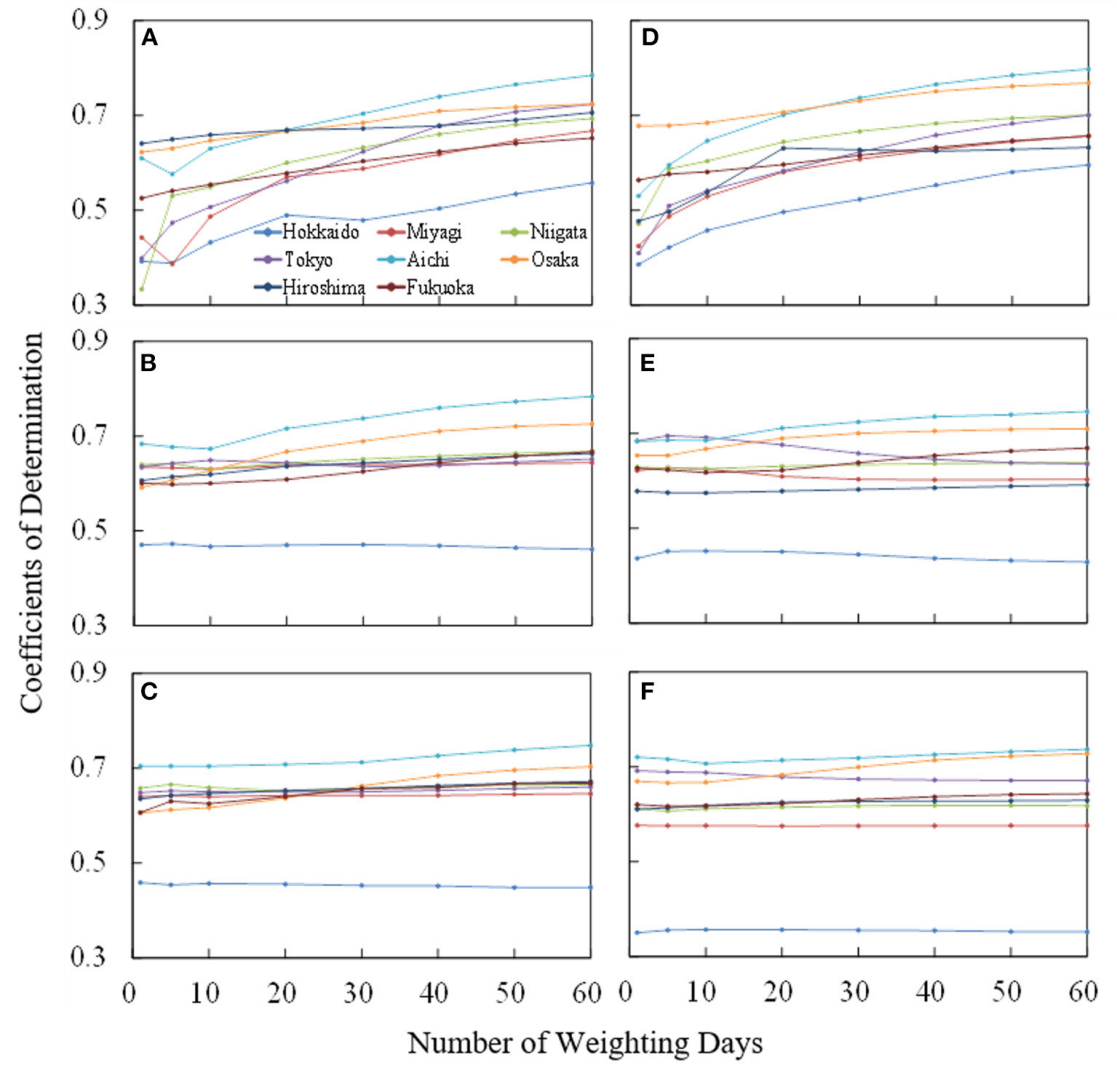
Variation of coefficient of determination averaged over 7 years (from June 1 to September 30 from 2013 to 2019) for the number of days over which an input variable is averaged, corresponding to *J* in Equation (4). For the number of patients transported the indoor locations, the **(A)** ambient temperature, **(B)** amount of sweating, and **(C)** body core temperature increase were considered. The same evaluation was conducted for patients transported from outdoor locations in **(D–F)**.

To clarify the duration that characterizes short-term heat adaptation, the correlation between observed and estimated numbers of patients transported from indoor/home and outdoor/workplace locations were evaluated in each prefecture for different input parameters. The evaluation period ran from June 1 to September 30 in 2013–2019. Figure 3 shows the coefficient of determination *R*^2^ averaged over the 7 years for the number of days over which an input variable is averaged, corresponding to *J* in Equation (4). The coefficients of determination of Hokkaido were lower than those of the remaining prefectures. The amount of sweating and core temperature increase were relatively small due to its milder climate. In the remainder of this report, our discussion focuses on the characteristics of the seven prefectures, excluding Hokkaido.

For the average ambient temperature, *R*^2^ increases with increase of averaging days (*J*) and then reached a plateau. The value of *R*^2^ is high in Tokyo, Aichi, and Osaka, where the population density is large. Even for the remaining prefectures, high coefficients of determination were observed. *R*^2^ reached a plateau at 40 days and 30 days of weighted days for the patients from indoor/home and outdoor/workplace locations, respectively.

The *R*^2^ in terms of computed core temperature and sweating were less sensitive to the averaged days than those for average ambient temperature, regardless of whether patients were transported from indoor/home or outdoor/workplace locations. Estimation accuracy was improved only in Aichi, Osaka, Hiroshima, and Fukuoka, where average summer temperatures are higher than in the other prefectures studied (see Table 1).

The optimal parameters for the equation for estimating the number of patients transported from indoor and outdoor locations are listed in Table 3. Note that the number of parameters used for fitting was four for ambient temperature and two for the core temperature and amount of sweating. The validity of the equations using the parameters in Table 3 is shown in Section 4 of the Supplementary material.

**Table 3:** The parameters in Equations (2–4) for estimating patients transported from indoor and outdoor locations for average ambient temperature, amount of sweating, and body core temperature increase.

**Input variables**		**Miyagi**	**Niigata**	**Tokyo**	**Aichi**	**Osaka**	**Hiroshima**	**Fukuoka**
**Indoor/home**								
Average temperature	*a*	3.55 × 10^−7^	7.01 × 10^−7^	2.01 × 10^−8^	2.83 × 10^−8^	3.05 × 10^−9^	2.49 × 10^−9^	2.44 × 10^−7^
	*l*	−3.07 × 10^3^	−2.51 × 10^3^	−2.33 × 10^4^	−3.08 × 10^4^	−1.09 × 10^4^	−2.14 × 10^−2^	−4.79 × 10^3^
	*f*	−8.25 × 10^−3^	−7.87 × 10^−3^	−6.97 × 10^−3^	−7.62 × 10^−3^	−3.90 × 10^−3^	−8.84 × 10^−3^	−5.41 × 10^−3^
	*g*	5.85 × 10^−1^	5.50 × 10^−1^	6.31 × 10^−1^	6.45 × 10^−1^	6.08 × 10^−1^	7.60 × 10^−1^	5.07 × 10^−1^
Amount of sweating	*a*	1.87 × 10^−1^	1.01	4.85 × 10^−2^	8.41 × 10^−3^	6.30 × 10^−3^	2.08 × 10^−2^	3.43 × 10^−1^
	*l*	−1.00	−1.00	−1.00	−1.00	−1.00	−1.00	−1.00
	*f*	−1.50 × 10^−7^	−2.04 × 10^−8^	−7.10 × 10^−8^	−3.42 × 10^−7^	−3.95 × 10^−7^	−1.94 × 10^−7^	−1.80 × 10^−8^
	*g*	2.01 × 10^−4^	2.72 × 10^−5^	2.34 × 10^−4^	1.01 × 10^−3^	1.09 × 10^−3^	5.74 × 10^−4^	4.00 × 10^−5^
Body core temperature increase	*a*	9.27 × 10^−3^	3.10 × 10^−2^	6.65 × 10^−3^	1.39 × 10^−3^	5.18 × 10^−4^	1.71 × 10^−3^	3.44 × 10^−3^
	*l*	−1.00	−1.00	−1.00	−1.00	−1.00	−1.00	−1.00
	*f*	−2.09 × 10^1^	−8.39	−5.22	−1.94 × 10^1^	−3.59 × 10^1^	−2.78 × 10^1^	−8.60
	*g*	8.97	3.18	5.28	1.30 × 10^1^	1.89 × 10^1^	1.47 × 10^1^	8.28
**Outdoor/workplace**								
Average temperature	*a*	1.21 × 10^−7^	8.36 × 10^−6^	3.42 × 10^−7^	1.85 × 10^−7^	2.30 × 10^−8^	5.25 × 10^−6^	4.31 × 10^−6^
	*l*	−5.13 × 10^3^	−4.55 × 10^2^	−3.92 × 10^3^	−9.62 × 10^3^	−2.50 × 10^3^	−2.99 × 10^2^	−7.42 × 10^2^
	*f*	−8.69 × 10^−3^	−6.40 × 10^−3^	−5.86 × 10^−3^	−6.78 × 10^−3^	−3.04 × 10^−3^	−2.22 × 10^−3^	−3.96 × 10^−3^
	*g*	6.35 × 10^−1^	4.38 × 10^−1^	5.19 × 10^−1^	5.76 × 10^−1^	5.34 × 10^−1^	3.34 × 10^−1^	3.88 × 10^−1^
Amount of sweating	*a*	1.00 × 10^2^	2.02 × 10^2^	2.02	8.03 × 10^−2^	2.85 × 10^−2^	7.76	4.70 × 10^1^
	*l*	−1.00	−1.00	−1.00	−1.00	−1.00	−1.00	−1.00
	*f*	N/A	N/A	N/A	N/A	N/A	N/A	N/A
	*g*	3.34 × 10^−7^	1.24 × 10^−7^	7.39 × 10^−6^	1.91 × 10^−4^	3.79 × 10^−4^	1.93 × 10^−6^	2.81 × 10^−7^
Body core temperature increase	*a*	7.94 × 10^−2^	2.76 × 10^1^	1.57 × 10^−2^	8.92 × 10^−3^	3.35 × 10^−3^	1.15 × 10^−2^	2.53 × 10^−2^
	*l*	−1.00	−1.00	−1.00	−1.00	−1.00	−1.00	−1.00
	*f*	N/A	N/A	N/A	N/A	N/A	N/A	N/A
	*g*	1.58	4.54 × 10^−3^	3.21	5.08	8.08	4.56	2.42

The parameters are derived for data from June 1 to September 30 from 2013 to 2019.

### 3.3. Estimation of heat illness morbidity in seven prefectures

**Figure 4:**
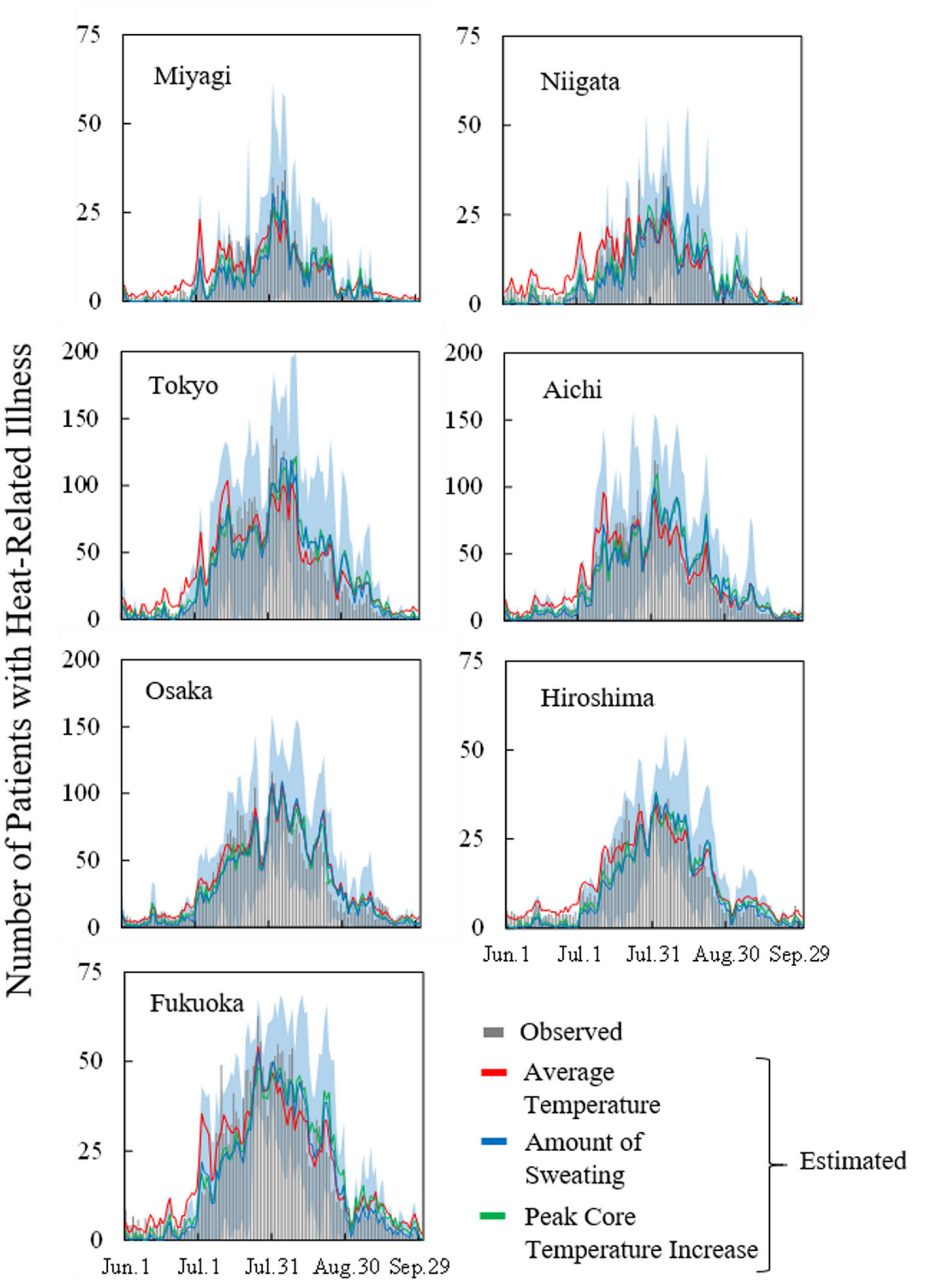
Observed and estimated number of patients with heat illness in seven prefectures (averaged over the period from 2013 to 2019) for average ambient temperature, amount of sweating, and body core temperature increase. Blue region represents the 95% confidence interval of estimation using computed daily amount of sweating.

Figure 4 shows the observed and estimated numbers of daily patients, averaged over 7 years, for each prefecture. Moreover, the confidence interval region (95%) when estimating with daily amount of sweating is also presented. *R*^2^ and MAEs per million population are listed in Table 4. The coefficient of determination *R*^2^ exceeded 0.6 and was particularly high for Tokyo, Aichi, and Osaka. No significant difference was observed in *R*^2^ between input parameters. MAEs have a mild correlation with the number of patients per million population. Because more patients were transported from outdoor locations, MAEs from those locations were larger than those from indoor locations. As seen in Figure 4, the estimated number of transported people is lower than the actual value and the variation is large just after the end of the rainy season (around July 20) in each prefecture, but actual value is almost within the 95% confidence interval. The number of transported patients was overestimated around early July for the average ambient temperature, while from mid-August to mid-September, the amount of sweating and body core temperature increase.

**Table 4:** Coefficient of determination *R*^2^ and MAEs per million population in seven prefectures (averaged over the period from 2013 to 2019).

	***R^2^*** **[All (Indoor/Outdoor)]**			**MAEs per million population [All (Indoor/Outdoor)]**		
	**Average temperature**	**Amount of sweating**	**Body core temperature increase**	**Average temperature**	**Amount of sweating**	**Body core temperature increase**
Miyagi	0.65 (0.62/0.61)	0.65 (0.64/0.58)	0.66 (0.64/0.61)	1.68 (0.77/1.02)	1.71 (0.76/1.07)	1.68 (0.77/1.01)
Niigata	0.70 (0.66/0.67)	0.66 (0.66/0.59)	0.68 (0.66/0.63)	1.97 (0.84/1.25)	1.95 (0.80/1.32)	1.90 (0.82/1.25)
Tokyo	0.68 (0.68/0.62)	0.71 (0.65/0.69)	0.69 (0.64/0.66)	1.25 (0.50/0.82)	1.11 (0.48/0.72)	1.20 (0.51/0.77)
Aichi	0.77 (0.74/0.74)	0.76 (0.73/0.71)	0.76 (0.76/0.68)	1.55 (0.61/1.05)	1.56 (0.61/1.12)	1.63 (0.57/1.22)
Osaka	0.74 (0.71/0.73)	0.71 (0.68/0.67)	0.74 (0.71/0.65)	1.47 (0.56/0.97)	1.55 (0.58/1.09)	1.48 (0.57/1.09)
Hiroshima	0.71 (0.68/0.63)	0.67 (0.66/0.61)	0.66 (0.65/0.57)	1.76 (0.76/1.21)	1.92 (0.81/1.22)	1.91 (0.81/1.25)
Fukuoka	0.64 (0.62/0.62)	0.67 (0.66/0.61)	0.66 (0.64/0.63)	1.77 (0.71/1.14)	1.67 (0.68/1.12)	1.74 (0.68/1.13)

**Figure 5:**
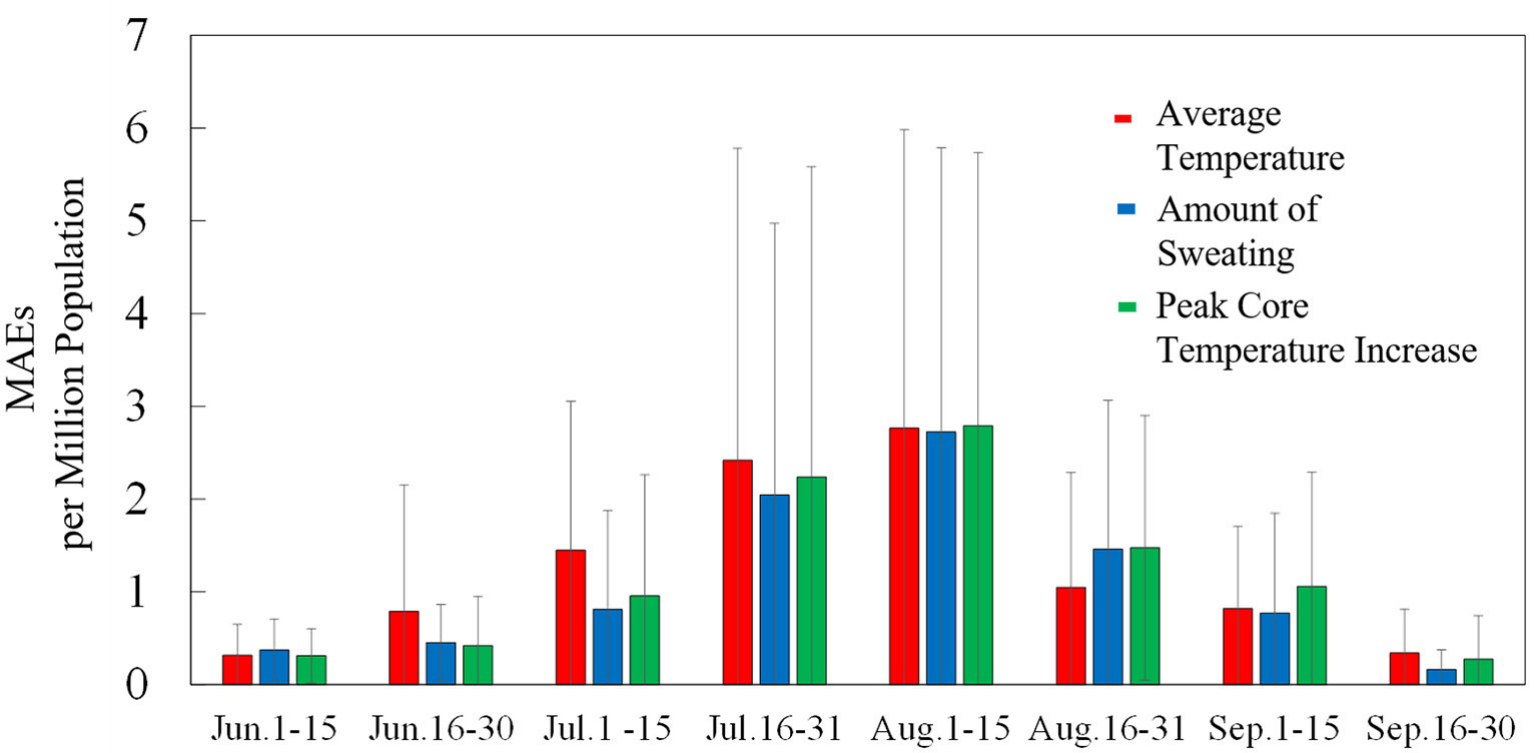
Time series of MAEs per million population in Tokyo for average ambient temperature, amount of sweating, and body core temperature increase (averaged over the period from 2013 to 2019). Standard deviations are indicated by error bars.

To clarify the findings from Figures 4, 5 shows the time series of MAEs averaged over the period from 2013 to 2019 in Tokyo. MAEs were greatest from mid-July to mid-August, when the number of patients was highest. Differences in estimation accuracy were seen among the input variables, as shown by the trends identified in Figure 4. In July, the estimation accuracy was high for the computed core temperature and sweating, and in late August, the accuracy was high for ambient temperature.

**Figure 6:**
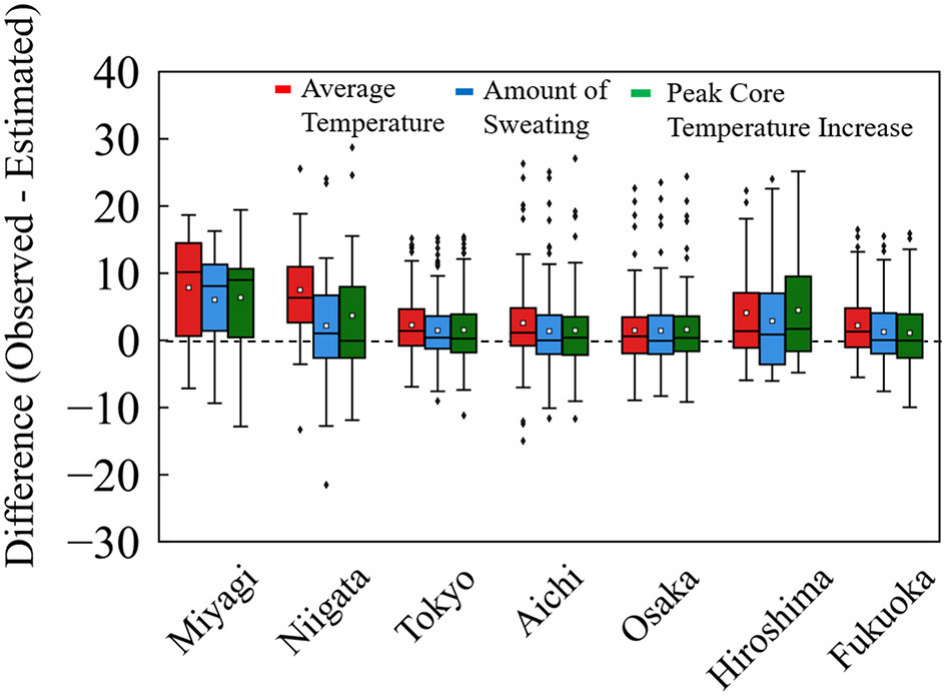
Difference from observed to estimated patients per million population in the days of WBGT ≥ 31°C. This approximately corresponds to a rate of transported patients per million population of ≥10 in seven prefectures from 2013 to 2019.

Ambulance allocation is crucial for days when the number of transported patients. To evaluate the effectiveness of the method of estimation for 3 months, we focus on accuracy where the ambient temperature is large. To calculate heat-related risk, wet-bulb global temperature (WBGT) (35) is often used, a value that takes into account humidity and solar radiation in addition to ambient temperature. In Tokyo, the daily average number of heat illness cases was 136 for WBGT ≥ 31°C. In Figure 6, we present a comparison of estimation accuracy among the input variables for WBGT ≥ 31°C. The criterion approximately corresponds to ≥10 transported patients per million population.

In Figure 6, comparable accuracy was observed for seven prefectures. The difference in MAE per million population, defined as observed value minus estimated value, was distributed on the negative side (overestimation) for computed core temperature and sweating, relative to that for average ambient temperature. Table 5 summarized the MAE per million population for the days of WBGT ≥ 31°C. From Table 5, for the amount of sweating and the increase in body core temperature, the values of MAE were marginally small, suggesting a better agreement for estimated and observed values for hot days with larger numbers of transported patients.

**Table 5:** Average MAEs per million population and number of days with WBGT ≥ 31°C for seven prefectures in Figure 6.

	**Average temperature**	**Amount of sweating**	**Body core temperature increase**	**Number of days**
Miyagi	9.75	7.84	9.26	14
Niigata	9.27	7.86	7.31	20
Tokyo	3.73	3.29	3.7	130
Aichi	4.45	4.46	4.32	102
Osaka	4.28	4.41	4.04	93
Hiroshima	5.89	6.35	6.55	29
Fukuoka	3.63)	3.71	4.03	173

## 4. Discussion

In this study, core temperature and sweating were computed to estimate the morbidity of heat illness patients for different prefectures of Japan. Eight prefectures were used that range from humid continental to subtropical climates (see Figure 1) (23).

In our analysis, Hokkaido, which belongs to Df in the Köppen climate classification scheme, was not well correlated with any input parameters because in that prefecture, body core temperature increase and sweating were low, even in August (see Figure 2). In other words, the number of hot days was limited, and thus, heat acclimatization is different or non-existent. One potential reason for this is that Hokkaido has a mild climate with only occasional heat waves. Thus, estimations covering very mild and extreme hot temperatures have less accuracy than those in the remaining prefectures. We then excluded Hokkaido for the remainder of the analysis.

As seen in Figure 2, the difference in daily peak core temperature and daily sweating amount were similar due to the daily course of ambient conditions. For the remaining seven prefectures, we derived parameters to estimate transported people in terms of Equations (2–4) with the parameters listed in Table 3. Note that the additional two parameters (*f* and *l*) were needed to obtain comparable accuracy in estimation of ambient temperatures to that shown in the sweating and core temperature.

As shown in Figure 3, the *R*^2^ reached plateau at 40 days and 30 days of weighted days for patients from indoor and outdoor locations, respectively. This takes into account the human acclimatization to heat in the environment for a certain period (a few weeks). The *R*^2^ values for computed core temperature and sweating were less sensitive to averaging days than that for average ambient temperature, regardless of whether patients were transported from indoor or outdoor locations. The *R*^2^ values for patients transported from outside is high even at 1 day (without averaging) or at least higher than the *R*^2^ for indoor patients with weighted average of < 20 days. This suggests that the effects of heat adaptation are not crucial for outdoor patients (especially for workers). This hypothesis indicates that heat acclimatization is not crucial for outdoor patients in reality, rather adjustments to behavior, such as changing clothing with changes in the season. For the indoor patients, some heat accumulation was observed for < 3 weeks. The numbers of patients transported from indoor and outdoor locations in Nagoya, Japan, were correlated with ambient temperature averaged over 50 and 20 days, respectively, in our previous study (20). The values obtained here are comparable to those data, from one city and one input variable.

Let us review heat accumulation further. Most previous studies investigated short-term adaptation *via* exercise (36–38). In Nakamura et al. (29), heat accumulation in daily life was investigated in a single region of Japan with a sample of five individuals, and it was found that 10 days were needed for intermittent heat exposure and 48 days for more continuous head adaptation. By contrast, this is a population-level study, and some difference can be observed (39, 40).

Smaller numbers of parameters were needed to estimate the numbers of transported people in terms of the computed thermophysiological responses, and these estimations provided better accuracy. An additional two parameters (*f* and *l*) were used in the estimation with ambient temperature, corresponding to heat accumulation (*f*) and additional tuning for accuracy compensation (*l*). Note that no physiological rationale exists for parameter (*l*) or a variable for different prefectures.

The estimated numbers of heat illness patients are in good agreement with observed values in seven prefectures. The proposed equations are applicable to different prefectures once weather, thermophysiological data, population, and age are incorporated. However, the estimation accuracy was variable for different seasons, as shown in Figure 5. In particular, the estimated numbers of people were smaller than the observed values following the rainy season, as shown in Figure 4. On those hot days, estimations with thermophysiological responses provided better accuracy (Figure 6).

The proposed equations have been used more than 20 times in the TV programs in Japan for public awareness. Fire departments in Nagoya City (Aichi prefectures) have introduced this system, sharing information with hospitals. The expected number of people transported owing to heat-related illness by using the proposed equations considering long-term temperature changes and the aging of the population in each region could be useful as a manner for future emergency systems. However, this system is tentatively suspended in COVID-19 epidemic.

This study was limited by the small number of extreme hot days included in the development of equations. Specifically, in 2018, the maximum ambient temperature was recorded in some prefectures. However, the estimation obtained on such days is an extrapolation, and thus accuracy is not warranted. Intense heat waves are expected to continue in the future, and thus the formula and parameters should also be revisited in the future.

## Data availability statement

Publicly available datasets were analyzed in this study. The details can be found in the article/Supplementary material.

## Author contributions

Conceptualization: AH, RE, MN, and SK. Methodology and formal analysis: AH, SK, and MN. Investigation and data curation: SK and AT. Resources and writing—review and editing: RE and HT. Writing original draft preparation: AT, SK, and AH. Project administration: AH, KS, and HT. All authors contributed to the article and approved the submitted version.
