# Supplementary material for: Estimation of the number of heat illness patients in eight metropolitan prefectures of Japan: Correlation with ambient temperature and computed thermophysiological responses
Source: Front Public Health. 2023 Feb 17;11:1061135. doi: 10.3389/fpubh.2023.1061135 (PMC9982159; doi:10.3389/fpubh.2023.1061135)
Supplement: Supplementary file 1 [file Data_Sheet_1.PDF]

Supplementary Material

1 Anatomical human model

A Japanese male model, which is based on magnetic resonance images, was used as a reference in this study (Nagaoka et al., 2004). The height and weight of this model were 1.73 m and 65 kg, respectively, which are close to those of mean values of Japanese males. This model consists of 2 mm cubic voxels (44,339,200 voxels) segmented into 51 anatomical tissues such as skin, fat, muscle, and bone. Figure S1 (A) illustrates the anatomical human model.

2 Body temperature computation

The body temperature were computed by the Pennes' bioheat transfer equation, which is expressed by the following equation (Pennes, 1948):

$$\boldsymbol{C}(\mathbf{r})\boldsymbol{\rho}(\mathbf{r})\frac{\partial \boldsymbol{T}(\mathbf{r},\boldsymbol{t})}{\partial \boldsymbol{t}} = \boldsymbol{\nabla}\cdot\left(\boldsymbol{K}(\mathbf{r})\boldsymbol{\nabla T}(\mathbf{r},\boldsymbol{t})\right) + \boldsymbol{M}(\mathbf{r},\boldsymbol{t}) + \boldsymbol{Q}(\mathbf{r},\boldsymbol{t}) - \boldsymbol{B}(\mathbf{r},\boldsymbol{t})\left(\boldsymbol{T}(\mathbf{r},\boldsymbol{t}) - \boldsymbol{T_B}(\boldsymbol{m},\boldsymbol{t})\right), \qquad (1)$$

where r and t denote three-dimensional position vector and time, T and T_B are the tissue temperature and the blood temperature in each body part (m = 1, …, 14) where m represents the body parts shown in Fig. 3 (b). The parameters C, K, B, M, and ρ denote the specific heat, thermal conductivity, a term associated with blood perfusion, a basal metabolism, and mass density of the tissue, respectively.

The boundary condition between the air and tissue were subjected to Eq. (2):

$$-\boldsymbol{K}(\mathbf{r})\frac{\partial \boldsymbol{T}(\mathbf{r},\boldsymbol{t})}{\partial \boldsymbol{n}} = \boldsymbol{H}(\mathbf{r},\boldsymbol{t})\cdot\left(\boldsymbol{T}(\mathbf{r},\boldsymbol{t}) - \boldsymbol{T_a}(\boldsymbol{t})\right) + \boldsymbol{EV}(\mathbf{r},\boldsymbol{t}), \quad (2)$$

where T_a is the ambient temperature. EV is evaporative heat loss, respectively. H is the heat transfer coefficient between air and tissue that combines the convective and radiative heat losses here (Fiala et al., 1999). The heat transfer coefficient varies according to surface and air temperature as same in (Kamiya et al., 2019). The specific heat, blood perfusion rate, heat conductivity and basal metabolism used herein are identical to those used in our previous study (Hirata et al., 2006). In addition, the blood perfusion rate through skin is the same in (McIntosh and Anderson, 2010).

In this study, the body core temperature was assumed to be equal to the blood temperature in the trunk. The time serious of blood temperature was computed considering arterial and venous temperatures for the body parts defined in Figure S1 (B). The arterial blood temperature was varied according to the following equation:

$$\boldsymbol{T_{bla,m}}(\boldsymbol{t}) = \frac{\boldsymbol{T_{blp}}(\boldsymbol{t})\sum_{\mathrm{r}}^{voxels}\boldsymbol{B}(\mathbf{r},\boldsymbol{t})\boldsymbol{V}(\boldsymbol{r})}{\boldsymbol{h_{x,m}} + \sum_{\mathrm{r}}^{voxels}\boldsymbol{B}(\mathbf{r},\boldsymbol{t})\boldsymbol{V}(\boldsymbol{r})} + \frac{\boldsymbol{h_{x,m}}\sum_{\mathrm{r}}^{voxels}\boldsymbol{T}(\mathbf{r},\boldsymbol{t})\boldsymbol{B}(\mathbf{r},\boldsymbol{t})\boldsymbol{V}(\boldsymbol{r})}{\sum_{\boldsymbol{r}}^{voxels}\boldsymbol{B}(\mathbf{r},\boldsymbol{t})\boldsymbol{V}(\mathbf{r})\left(\boldsymbol{h_{x,m}} + \sum_{\mathrm{r}}^{voxels}\boldsymbol{B}(\mathbf{r},\boldsymbol{t})\boldsymbol{V}(\mathbf{r})\right)}, \qquad (3)$$

where $T_{bla,m}$ denotes the arterial and venous temperatures in each body part m, respectively. The blood temperatures in the head and torso were assumed to be same due to their high blood perfusion rate (Fiala et al., 1999). The values of V, h_x, and m are given in (Kamiya et al., 2019).

The vasodilatation according to temperature rise above a certain level were considered as the same as in (Laakso and Hirata, 2011).

The evaporative heat loss from the skin is given as follows (Stolwijk, 1971):

$$\boldsymbol{EV}(\mathbf{r},\boldsymbol{t}) = \boldsymbol{min}\{\boldsymbol{SW}(\mathbf{r},\boldsymbol{t})\cdot\mathbf{40.6}/\boldsymbol{S},\boldsymbol{EV}_{\boldsymbol{max}}(\boldsymbol{t})\},$$
$$\boldsymbol{EV}_{\boldsymbol{max}}(\boldsymbol{t}) = \mathbf{2.2}\cdot\boldsymbol{h}_{\boldsymbol{c}}\boldsymbol{f}_{\boldsymbol{pcl}}\left(\boldsymbol{P}_{\boldsymbol{s}}(\boldsymbol{t}) - \boldsymbol{\varphi}_{\boldsymbol{\varepsilon}}\boldsymbol{P}_{\boldsymbol{A}}(\boldsymbol{t})\right), \qquad (\mathbf{4})$$

where SW is the sweating rate [g/min], S [m^2] is the total surface area of the human body, and 40.6 J/g is a conversion coefficient. The maximum evaporative heat loss, EV_{max}, on the skin depends on the ambient conditions. The convective heat transfer coefficient is represented by h_c; P_S and P_A are the saturated water vapor pressures at the temperature of the skin and at the ambient air temperature, respectively; φ_e is the relative humidity of the ambient air, and f_{pcl} is the permeation efficiency factor of clothing, which is affected by the wind velocity. For simplicity, f_{pcl} is assumed to be 0.8, corresponding to a subject wearing short sleeves and long pants (Nishi and Gagge, 1970; Laakso and Hirata, 2011).

The evaporative heat loss in elderly was modeled considering decline in skin perception and sweat gland (Hirata et al., 2012, 2014). There is no information on the age category when the data is divided into indoor and outdoor. Therefore, a thermoregulation model assuming adult males was applied to computed core temperature and perspiration.

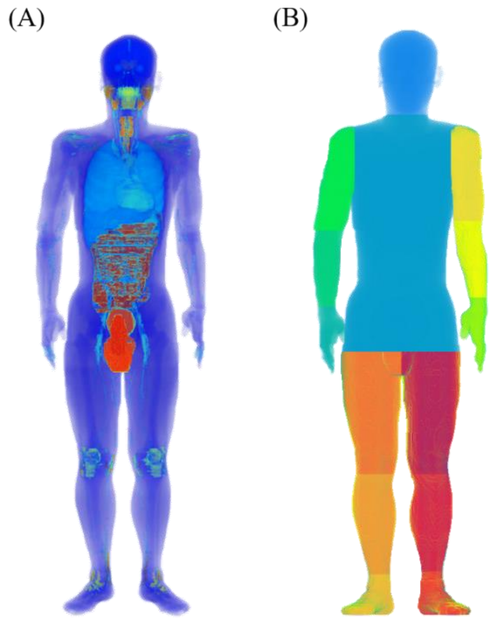

Figure S1. (A) Anatomical human model of adult male and **(B)** definition of body parts, representing a test subject.

3 Weather conditions in eight prefectures

Figure S2 shows the daily average and maximum ambient temperatures for each prefecture in 2019 (Japan Meteorological Agency, 2020), used to compute core temperature increase and sweating shown in Figure 2. As shown in Table 1, temperatures were lowest in Hokkaido and higher as one moves southward. The highest temperatures were observed in early August in each prefecture, and similar tendencies were observed in the computed body core temperature and sweating amount in Figure 2. The correlation coefficients between the average ambient temperature and the calculated results (body core temperature increase and sweating amount) were about 0.87 (p<0.05) and 0.8 (p<0.05), respectively, for the average of eight prefectures.

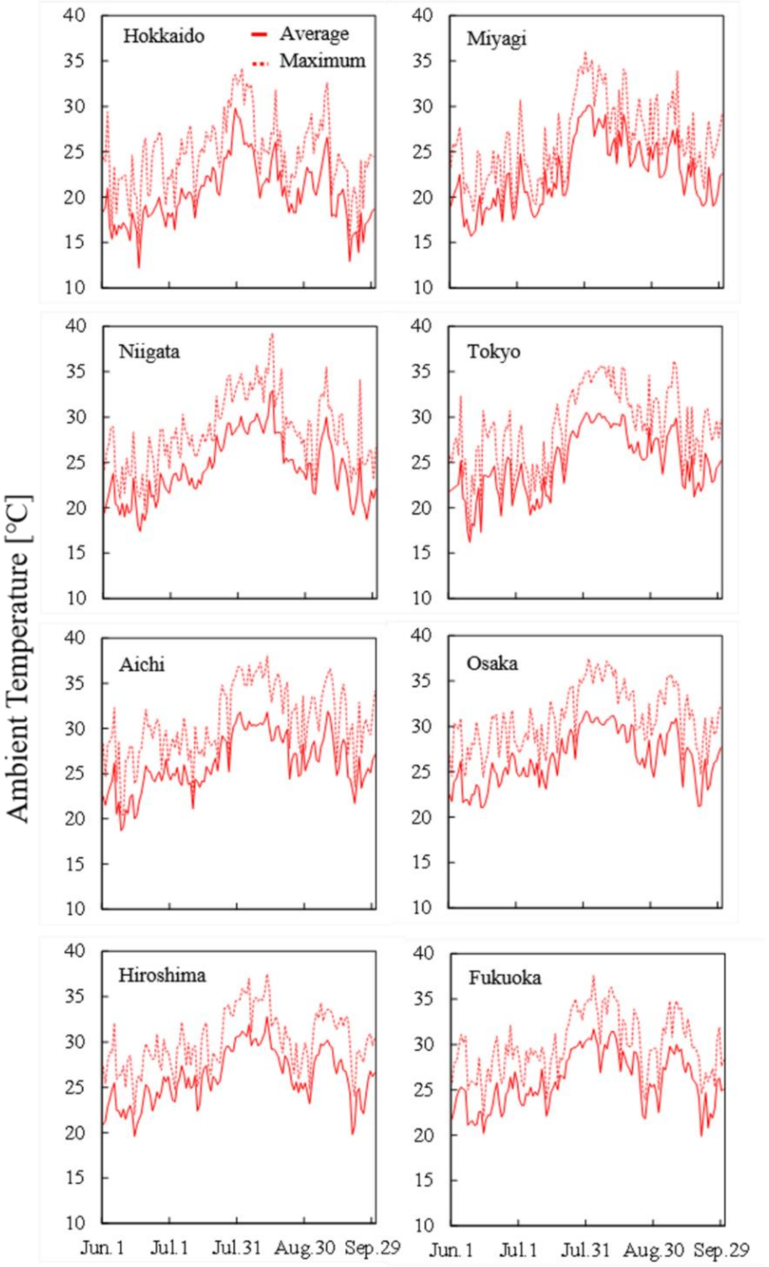

Figure S2. Time course of daily average and maximum ambient temperatures in each prefecture in 2019.

4 Evaluation of the validity of the regression model

The F-test was carried out evaluate statistical significance of the proposed equations. Table S1 shows the F-values when the regression parameters were set in Table 2. As shown in Table S1, the proposed equation was statistically significant for all input variables and prefectures.

Table S1. F-values for regression model with parameters in Table 2.

Prefectures	Classification	Average Temperature	Amount of sweating	Body core temperature increase
Miyagi	Indoor	512.7^{***}	621.0^{***}	547.5^{***}
	Outdoor	732.1^{***}	1827.7^{***}	1869.5^{***}
Niigata	Indoor	424.0^{***}	629.0^{***}	534.8^{***}
	Outdoor	792.7^{***}	1898.0^{***}	1971.9^{***}
Tokyo	Indoor	475.5^{***}	536.5^{***}	450.6^{***}
	Outdoor	757.0^{***}	2545.1^{***}	2015.3^{***}
Aichi	Indoor	602.5^{***}	755.2^{***}	872.1^{***}
	Outdoor	1278.0^{***}	2565.6^{***}	1981.8^{***}
Osaka	Indoor	552.2^{***}	570.6^{***}	608.7^{***}
	Outdoor	1205.1^{***}	2269.0^{***}	1988.2^{***}
Hiroshima	Indoor	683.3^{***}	702.5^{***}	667.5^{***}
	Outdoor	626.2^{***}	1786.3^{***}	1373.8^{***}
Fukuoka	Indoor	370.1^{***}	488.6^{***}	425.5^{***}
	Outdoor	677.6^{***}	1797.7^{***}	1626.5^{***}

*$p<0.05$, **$p<0.01$, ***$p<0.001$

5 Reference

Fiala, D., Lomas, K. J., and Stohrer, M. (1999). A computer model of human thermoregulation for a wide range of environmental conditions: the passive system. *J. Appl. Physiol.* 87, 1957–1972. doi:10.1152/jappl.1999.87.5.1957.

Hirata, A., Fujiwara, O., and Shiozawa, T. (2006). Correlation between peak spatial-average SAR and temperature increase due to antennas attached to human trunk. *IEEE Trans. Biomed. Eng.* 53, 1658–1664. doi:10.1109/TBME.2006.877798.

Hirata, A., Laakso, I., Ishii, Y., Nomura, T., and Chan, K. H. (2014). Computation of temperature elevation in a fetus exposed to ambient heat and radio frequency fields. *Numer. Heat Transf. Part A Appl.* 65, 1176–1186. doi:10.1080/10407782.2013.869075.

Hirata, A., Nomura, T., and Laakso, I. (2012). Computational estimation of decline in sweating in the elderly from measured body temperatures and sweating for passive heat exposure. *Physiol. Meas.* 33. doi:10.1088/0967-3334/33/8/N51.

Japan Meteorological Agency (2020). Wether database (in Japanese).

Kamiya, T., Kodera, S., Hasegawa, K., Egawa, R., Sasaki, H., and Hirata, A. (2019). Different thermoregulatory responses of people from tropical and temperate zones: A computational study. *Build. Environ.* 159, 106152. doi:10.1016/j.buildenv.2019.05.030.

Laakso, I., and Hirata, A. (2011). Dominant factors affecting temperature rise in simulations of human thermoregulation during RF exposure. *Phys. Med. Biol.* 56, 7449–7471. doi:10.1088/0031-9155/56/23/008.

McIntosh, R. L., and Anderson, V. (2010). A comprehensive tissue properties database provided for the thermal assessment of a human at rest. *Biophys. Rev. Lett.* 05, 129–151. doi:10.1142/S1793048010001184.

Nagaoka, T., Watanabe, S., Sakurai, K., Kunieda, E., Watanabe, S., Taki, M., et al. (2004). Development of realistic high-resolution whole-body voxel models of Japanese adult males and females of average height and weight, and application of models to radio-frequency electromagnetic-field dosimetry. *Phys. Med. Biol.* 49, 1–15. doi:10.1088/0031-9155/49/1/001.

Nishi, Y., and Gagge, A. P. (1970). Moisture permeation of clothing : A factor governing thermal equilibrium and comfort. *ASHRAE Trans.* 76, 137–145.

Pennes, H. H. (1948). Analysis of tissue and arterial blood temperatures in the resting human forearm. *J. Appl. Physiol.* 1, 93–122.

Stolwijk, J. A. (1971). A mathematical model of physiological temperature regulation in man. *NASA Contract. Rep.* NASA CR-18, 77. doi:NASA CR-1855.
